# Antimicrobial Activity of Serbian Propolis Evaluated by Means of MIC, HPTLC, Bioautography and Chemometrics

**DOI:** 10.1371/journal.pone.0157097

**Published:** 2016-06-07

**Authors:** Petar Ristivojević, Ivica Dimkić, Jelena Trifković, Tanja Berić, Irena Vovk, Dušanka Milojković-Opsenica, Slaviša Stanković

**Affiliations:** 1 Innovation Centre of the Faculty of Chemistry Ltd., University of Belgrade, Belgrade, Serbia; 2 Department of Microbiology, Faculty of Biology, University of Belgrade, Belgrade, Serbia; 3 Department of Analytical Chemistry, Faculty of Chemistry, University of Belgrade, Belgrade, Serbia; 4 Laboratory for Food Chemistry, National Institute of Chemistry, Ljubljana, Slovenia; College of Agricultural Sciences, UNITED STATES

## Abstract

New information has come to light about the biological activity of propolis and the quality of natural products which requires a rapid and reliable assessment method such as High Performance Thin-Layer Chromatography (HPTLC) fingerprinting. This study investigates chromatographic and chemometric approaches for determining the antimicrobial activity of propolis of Serbian origin against various bacterial species. A linear multivariate calibration technique, using Partial Least Squares, was used to extract the relevant information from the chromatographic fingerprints, *i*.*e*. to indicate peaks which represent phenolic compounds that are potentially responsible for the antimicrobial capacity of the samples. In addition, direct bioautography was performed to localize the antibacterial activity on chromatograms. The biological activity of the propolis samples against various bacterial species was determined by a minimum inhibitory concentration assay, confirming their affiliation with the European poplar type of propolis and revealing the existence of two types (blue and orange) according to botanical origin. The strongest antibacterial activity was exhibited by sample **26** against *Staphylococcus aureus*, with a MIC value of 0.5 mg/mL, and *Listeria monocytogenes*, with a MIC as low as 0.1 mg/mL, which was also the lowest effective concentration observed in our study. Generally, the orange type of propolis shows higher antimicrobial activity compared to the blue type. PLS modelling was performed on the HPTLC data set and the resulting models might qualitatively indicate compounds that play an important role in the activity exhibited by the propolis samples. The most relevant peaks influencing the antimicrobial activity of propolis against all bacterial strains were phenolic compounds at *R*_F_ values of 0.37, 0.40, 0.45, 0.51, 0.60 and 0.70. The knowledge gained through this study could be important for attributing the antimicrobial activity of propolis to specific chemical compounds, as well as the verification of HPTLC fingerprinting as a reliable method for the identification of compounds that are potentially responsible for antimicrobial activity. This is the first report on the activity of Serbian propolis as determined by several combined methods, including the modelling of antimicrobial activity by HPTLC fingerprinting.

## Introduction

Propolis is a natural sticky substance collected by honeybees (*Apis mellifera* L.) from the buds of numerous plant species, depending on the climate zone. It is used by honeybees as glue, to fill cracks in hives, and as a protective barrier against intruders such as microbes, snakes, mice, etc. [1,2]. Propolis is in no way a new discovery. Extensive employment of propolis as an antiseptic and antipyretic dates back to ancient times in many cultures, including the Egyptians, Incas, Greeks, Romans, and Slavs. Propolis has also been recognized as an official drug in London pharmacopeias since the 17^th^ century, and it is still one of the most frequently used natural remedies in the Balkan States [2]. It has only been in the last decades that scientists have investigated its constituents and its biological properties with regard to its ethnopharmacological use, e.g. its antiseptic and immunomodulatory properties. The ethnopharmacological approach, combined with chemical and biological methods, may provide useful pharmacological leads [2]. The chemical composition of propolis depends on its geographical origin, local flora, the species of bee, and the season [3]. Generally, propolis is composed of 50% plant resin, 30% wax, 10% essential and aromatic oils, 5% pollen and 5% various other substances [1]. One of the functions of propolis in the beehive is to protect it against microbial invaders. The antimicrobial effects of propolis are well documented against various bacteria, yeasts, viruses and parasites [2,4].

Several pharmacological properties of propolis, mainly it’s antibacterial, antiviral, antifungal, anti-inflammatory, and antioxidant properties, have been attributed to phenolic compounds [2–4]. Analytical techniques such as ultraviolet/visible spectroscopy, thin-layer chromatography (TLC), electrophoresis, high-performance liquid chromatography (HPLC), high-performance thin-layer chromatography (HPTLC) and different mass spectrometry techniques have been developed for the analysis of phenolic compounds found in propolis samples [5,6]. The phenolic profile of Serbian propolis was analyzed in detail by HPTLC and ultra-high performance liquid chromatography—mass spectrometry (UHPLC–LTQ OrbiTrap MS/MS) using multivariate image analysis and pattern recognition methods, revealing the existence of two varieties, orange and blue, characteristic of propolis made by bees foraging on European poplar trees [7–9].

Multivariate modelling techniques can be used to obtain information from spectral data. Different linear multivariate calibration techniques, such as multiple linear regression (MLR), principal component regression (PCR) and partial least square regression (PLS), have been described in the literature for predicting the antioxidative activity of green tea [10] and *Mallotus* species [11], and the lymphocyte proliferation capacity of *Panax ginseng* [12], among other experiments. In addition, a microbiological screening method hyphenated with planar chromatography techniques, called bioautography, is commonly used for the identification of bioactive compounds present in crude extracts [13].

This article is a continuation of a comprehensive assessment of propolis samples from Serbia [7,8] as a contribution to the characterization of European poplar propolis. The aim of this study is to evaluate the utility of modelling the antimicrobial activity of these propolis samples using HPTLC phenolic fingerprints, not in order to predict their biological activity, but to indicate peaks, *i*.*e*. compounds potentially responsible for antibacterial capacity. Until now, there have been no publications on the modelling of antimicrobial activity using HPTLC fingerprints. The antimicrobial effect on bacterial species was first determined in a minimum inhibitory activity (MIC) assay. In addition, direct bioautography was performed to localize antibacterial activity on chromatograms. To the best of our knowledge, this is also the first report on the antimicrobial activity of Serbian propolis as determined by several combined methods.

## Materials and Methods

### Chemicals and materials

2-Aminoethyl diphenylborinate (NTS) was purchased from Fluka (Steinheim, Germany), toluene and ethyl acetate from Merck (KGaA, Darmstadt, Germany), polyethyleneglycol (PEG) and methanol from Sigma-Aldrich (Steinheim, Germany), and formic acid from Kemika (Zagreb, Croatia). Phenolic standards such as quercetin, apigenin, kaempferol, rutin, myricetin, chrysin, luteolin, pinostrombin, pinobanksin, and naringenin were purchased from Fluka AG (Buch, Switzerland), while *p*-coumaric, caffeic, ferulic acid, and gallic acids were supplied by Sigma-Aldrich (Steinheim, Germany). Syringe filters (13 mm, PTFE membrane 0.45 μm) were purchased from Supelco (Bellefonte, PA, USA). In this study, a aqueous resazurin solution (0.675 mg/mL final concentration) of Resazurin Sodium Salt C_12_H_6_NNaO_4_ (TCI, Belgium) and a 3-(4,5-dimethylthiazol-2-yl)-2,5-diphenyltetrazolium bromide (MTT) solution of 0.2% Thiazolyl Blue Tetrazolium Bromide C_18_H_16_BrN_5_S (Sigma-Aldrich, USA) with 0.1% Triton X-100 (C_14_H_22_O (C_2_H_4_O)_*n*_) (Sigma-Aldrich, USA) were used.

### Propolis samples

A total of 53 propolis samples were collected from different regions of Serbia during 2010 and 2011 using the scraping harvesting method and stored at −20°C prior to analysis. All samples were collected from the hives of *Apis mellifera* L. Information about the propolis samples, geographical coordinates, wax content and classification according to the HPTLC phenolic profile, is presented in S1 Table. Methanolic extracts of propolis samples were prepared according to the method described previously [7]. We confirmed that no specific permissions were required by authorities for the locations or activities involved. The beekeepers willingly provided the samples of propolis for this study. We also confirmed that the scope of our study did not involve endangered or protected species.

### Bacterial strains and growth conditions

Antibacterial activity was tested using two Gram negative strains: (i) *Aeromonas hydrophila* (ATCC 49140) and *Shigella flexneri* (ATCC 9199); and a panel of Gram positive strains: (ii) *Listeria monocytogenes* ATCC 19111, *Bacillus subtilis* ATCC 6633, *Enterococcus faecalis* ATCC 29212, and *Staphylococcus aureus* ATCC 25923. The initial screening of antibacterial activity was performed using five more Gram negative strains: (i) *Enterobacter cloacae* (ATCC 49141), *Escherichia coli* (ATCC 25922), *Proteus mirabilis* (ATCC 25933), *Pseudomonas aeruginosa* (ATCC 15422) and *Salmonella enteritidis* (ATCC 13076); and two more Gram positive strains: (ii) *Micrococcus luteus* (ATCC 7468) and *Streptococcus equisimilis* (ATCC 12394). The bacterial strains were cultured overnight at 37°C in MHB (Müller-Hinton broth, HiMedia, India) with the exception of *L*. *monocytogenes* which was cultured in BHI broth (Brain-Heart Infusion, Biomedics, Spain). Suspensions were adjusted to McFarland standard turbidity (0.5) which corresponds to 10^7^–10^8^ CFU/mL.

### Antibacterial assays

The initial screening of antibacterial activity of propolis samples was performed by disc-diffusion assay against thirteen Gram positive and Gram negative bacteria. Petri dishes with MHA (Müller-Hinton agar) solid medium were poured with 5 mL of soft MHB medium, previously inoculated with 50 μL of particular indicator strain. Disc-diffusion assay was completed by adding tested propolis in different concentrations (1.5–0.01 mg/disc) onto the 5 mm disc (Abtek Biologicals, England). Petri dishes were incubated for 24 h at 37°C. The results were obtained by measuring the diameter of growth inhibition zone and expressed in mm. All experiments are performed as triplicates.

A broth microdilution method was used to determine the minimum inhibitory concentrations and minimum bactericidal concentrations (MBC) of the tested samples of propolis. Final concentrations of propolis samples in the first well ranged from 7–20 mg/mL, depending on the sample. The final concentration of methanol as a solvent was 10%. Two-fold serial dilutions with MHB medium in 96-well microtiter plates were performed, with the exception of *L*. *monocytogenes*, for which BHI medium was used. Besides a negative control, a sterility control, and a control for the solvent (methanol), the antibiotics streptomycin, ampicillin and rifampicin were also tested as positive controls. All dilutions were done in duplicate. Each well, except for the sterility control, was inoculated with 20 μL of bacterial culture (approx. 1 × 10^8^ CFU/mL), reaching a final volume of 200 μL. At the end, 22 μL of resazurin was added to each well. The plates were incubated for 24 h at 37°C. All tests were performed in a lighted environment, but the plates were incubated in the dark. Resazurin is an oxidation-reduction indicator used for the evaluation of cell growth. It is a blue non-fluorescent and non-toxic dye that becomes pink and fluorescent when reduced to resorufin by oxidoreductases within viable cells [14]. The lowest concentration which showed no change in colour was defined as the MIC. MBC was determined by sub-culturing the test dilutions from each well without colour change on agar plates and incubating for 18–24 h. The lowest concentration that didn’t show bacterial growth was defined as the MBC value. The results are expressed in mg/mL.

### High-performance thin-layer chromatography and bioautography

The HPTLC method used for obtaining phenolic profiles of the propolis samples was described previously [7]. Simultaneously, phenolic standards were chromatographed under the same conditions (S1 Fig).

For preparing the HPTLC plates for bioautography, 2μL of each propolis extract was applied to 10 cm × 10 cm HPTLC Silica gel 60 F_254_ glass plates (Art. 5641, Merck, Darmstadt, Germany) in an 8 mm band by using an Automatic TLC sampler 4 (ATS4, CAMAG, Muttenz, Switzerland). Development was performed to a distance of 8 cm in a horizontal developing chamber (CAMAG) using 6 mL a mixture of *n*-hexane-ethyl acetate-acetic acid (5:3:1, v/v/v). The developed plates were dried in a stream of warm air, followed by an agar overlay variant of bioautography, a modified method of Valgas et al. [15]. Autoclave tape was put on the bottom of the plate around the edges, in that way forming a mold, 10 × 10 cm, 0.5 cm deep. All HPTLC plates with samples were sterilized for 15 minutes under UV light. MHB or BHI soft agar (7 mL), previously inoculated with 70 μL (1 × 10^8^ CFU/mL) of tested bacteria, was spread on the plates. HPTLC silica gel 60 plates, 20 × 10 cm, were placed in plastic boxes, with wet cotton balls for keeping the atmosphere humid during incubation, for 24 h at 37°C in the dark. After incubation, the plates were sprayed with 0.2% MTT for the visualization of inhibition zones, and then incubated for 3–4 h.

### Data acquisition and statistical analysis

Images of the plates were processed with the ImageJ processing program (http://imagej.nih.gov/ij/ver. 1.47q, Rasband W. National Institutes of Health, USA). All data alignment and image analysis techniques were described in our previous paper [7]. Partial least squares regression (PLS) was carried out by means of PLS Toolbox v.6.2.1, from MATLAB 7.12.0 (R2011a). PLS is a regression multivariate method that calculates new latent variables (LVs) for independent (X) and dependent (Y) variable matrices and a relationship between them. Latent variables show high variation, highly correlated with Y, and their optimal number is usually determined by cross-validation. The contribution of the variables to the final model can be interpreted by evaluating their regression coefficient [10].

## Results and Discussion

HPTLC phenolic fingerprinting of Serbian propolis samples revealed the existence of two main botanically distinct types of propolis, an orange type characterized by intense orange and blue bands and pale green zones, and a blue type showing a chemical profile full of blue bands, confirmed by the application of different pattern recognition techniques [7]. The orange propolis samples showed identical chromatographic profiles, contrary to the blue type whose phenolic composition varies between samples. It was also observed that orange was the prevailing type.

### Antimicrobial activity of propolis extracts

The antimicrobial activity of the Serbian propolis samples was tested against thirteen bacterial strains, initially. Disk diffusion method was used for primary screening. Sensitivity of propolis samples toward seven Gram negative bacteria (*A*. *hydrophila*, *E*. *cloacae*, *E*. *coli*, *P*. *mirabilis*, *P*. *aeruginosa*, *S*. *enteritidis* and *S*. *flexneri*) and six Gram positive bacteria (*B*. *subtilis*, *E*. *faecalis*, *S*. *equisimilis*, *M*. *luteus*, *S*. *aureus* and *L*. *monocytogenes*) was tested.

Of all analyzed propolis (data not shown) the strongest antimicrobial effect against *A*. *hydrophila* and *S*. *flexneri* showed propolis samples **10**, **17**, **28**, **29** and **30**, with the appearance of zones of inhibition already at 0.05 mg/disc, while at a concentration of 0.20 mg/disc, their diameter was larger than 12 mm. The strongest activity against most Gram positive bacteria was achieved by samples: **7**, **8**, **9**, **17**, **19**, **28**, **29**, **32** and **51**. The lowest active MIC was 0.01 mg/disc, especially against *L*. *monocytogenes*, *S*. *aureus*, *E*. *faecalis*, and *B*. *subtilis*. The strongest effect of most tested propolis has been observed against *B*. *subtilis* and *L*. *monocytogenes*, with a zone of inhibition greater than 12 mm, at a concentration from 0.15 mg/disc. Propolis samples **1**, **2**, **3**, **4**, and **6** showed moderate antimicrobial activity against all tested strains (zones of inhibition were between 8–12 mm) starting from 0.01 mg/disc against certain Gram positive, i.e., 0.10 mg/disc for Gram negative bacteria. An *E*. *cloacae* was sensitive only to these five samples. Propolis samples **9**, **11**, **12**, **15**, **16**, **17**, **19**, **28**, **29**, **30**, **32** and **51** failed to inhibit *E*. *coli*, *P*. *mirabilis*, *P*. *aeruginosa* and *S*. *enteritidis*, at any concentration. Towards *E*. *coli* activity was detected only with samples **7**, **8** and **10**, at higher concentrations (0.20–1.5 mg/disc). Similar activities for samples **7**, **8** and **10** were obtained against *M*. *luteus* and *S*. *equisimilis*, which otherwise, were the most resistant among Gram positive strains. Propolis samples **11**, **12**, **15**, **16**, **17**, **19**, **28**, **29**, **30**, **32** and **51** had no effect against *M*. *luteus*, while samples **11** till **19** were active against *S*. *equisimilis* only at higher concentrations.

The MIC and MBC values of 53 propolis samples were determined for each chosen bacteria. As indicator strains we used those that showed the highest sensitivity in primary screening: *B*. *subtilis*, *E*. *faecalis*, *S*. *aureus*, *L*. *monocytogenes*, *A*. *hydrophila* and *S*. *flexneri*. The MIC values for the propolis samples are shown in Table 1 as well as MBC values in S2 Table. Although spectrometric reading at a specific wavelength is a more objective and quantifiable method, due to the continuous sedimentation of samples and a failure to get correct readings resazurin reaction was used for determining the antimicrobial activity of propolis instead. Methanol was used in the first well as a negative control for each indicator strain and it did not show any effect on growth at the final concentration (10%).

**Table 1:** The Minimum Inhibitory Concentration (MIC) of Methanol Extracts of Serbian Propolis Samples in mg/mL.

Extracts of propolis	Type of propolis	*Aeromonas hydrophila*ATCC 49140	*Shigella flexneri* ATCC 9198	*Listeria monocytogenes* ATCC 19111	*Staphylococcus aureus*ATCC 25922	*Bacillus subtilis* ATCC 6632	*Enterococcus faecalis*ATCC 29212
2	**O**	3.0	6.1	0.4*	3.0	1.5	6.1
3	**O**	3.4	6.9	**0.9**	13.7	3.4	**0.9**
6	**O**	6.6	3.3	**0.8**	13.3	3.3	1.7
7	**O**	13.4	7.4	1.9	7.4	14.9	1.9
8	**O**	15.1	7.5	1.9	10.5	3.8	1.9
9	**O**	12.9	7.2	**0.9**	7.2	3.6	1.8
10	**O**	7.3	1.8	**0.9**	14.5	1.8	**0.9**
11	**O**	14.1	3.5	**0.4**	7.1	**0.4**	**0.9**
12	**O**	12.4	12.4	1.5	12.4	**0.8**	6.2
13	**O**	3.8	7.5	**0.9**	**0.5**	**0.9**	**0.9**
14	**O**	3.6	3.6	**0.9**	**0.4**	**0.9**	1.8
16	**O**	12.3	3.1	**0.8**	6.1	**0.4**	1.5
17	**O**	5.5	1.4	**0.3**	5.5	**0.3**	**0.3**
19	**O**	13.7	6.9	**0.9**	13.7	**0.4**	3.4
20	**O**	1.6	6.4	**0.8**	**0.4**	**0.8**	1.6
21	**O**	3.1	6.1	**0.8**	**0.4**	**0.4**	1.5
22	**O**	7.3	14.7	**0.9**	**0.5**	**0.9**	3.7
24	**O**	1.5	3.9	1.0	**0.5**	1.0	1.9
25	**O**	1.1	3.5	1.8	**0.4**	**0.4**	1.8
26	**O**	1.9	1.0	**0.1**	**0.5**	1.0	2.0
27	**O**	8.7	8.7	2.2	1.1	2.2	4.4
28	**O**	3.9	3.9	**0.5**	7.9	2.0	15.8
29	**O**	5.8	2.9	**0.4**	11.6	2.9	11.6
30	**O**	8.0	8.0	**0.5**	16.1	8.0	1.0
31	**O**	1.4	2.4	**0.9**	0.6	**0.6**	2.4
32	**O**	4.7	4.7	**0.3**	9.4	2.4	1.2
33	**O**	**0.9**	3.5	**0.9**	**0.4**	**0.4**	**0.4**
34	**O**	**0.3**	3.5	**0.3**	**0.4**	**0.4**	**0.4**
36	**O**	3.6	7.1	**0.4**	**0.4**	**0.9**	**0.4**
37	**O**	4.5	9.0	**0.4**	1.1	2.3	2.3
38	**O**	7.5	15.0	**0.3**	**0.5**	**0.9**	1.9
40	**O**	3.4	8.5	**0.4**	**0.5**	1.1	2.1
41	**O**	**0.9**	3.0	**0.8**	**0.4**	**0.4**	1.5
42	**O**	1.2	3.9	1.0	**0.5**	1.0	1.9
43	**O**	1.0	5.1	**0.6**	**0.3**	**0.6**	1.3
44	**O**	**0.5**	8.9	1.1	**0.6**	2.2	2.2
45	**O**	1.0	6.9	**0.9**	**0.4**	**0.9**	1.7
46	**O**	**0.3**	8.4	1.1	**0.5**	1.1	2.1
47	**O**	3.3	8.3	**0.4**	**0.5**	2.1	2.1
49	**O**	3.4	14.4	**0.2**	1.1	1.1	2.1
50	**O**	4.6	10.9	**0.4**	**0.5**	1.0	1.9
51	**O**	16.8	8.4	1.1	4.2	8.4	16.8
1	**B**	3.9	4.6	1.9	3.9	3.9	3.9
4	**B**	7.1	7.1	**0.9**	14.2	7.1	1.8
5	**B**	14.5	14.5	1.8	14.5	7.2	3.6
15	**B**	12.9	12.9	6.5	12.9	3.2	12.9
18	**B**	3.2	6.3	3.2	**0.8**	1.6	3.2
23	**B**	6.8	13.5	**0.4**	1.7	13.5	3.4
35	**B**	3.3	8.2	4.1	4.1	8.2	8.2
39	**B**	5.0	10.0	2.4	1.3	2.5	5.0
48	**B**	4.8	10.6	10.6	5.3	10.6	10.6
52	**B**	7.2	7.9	1.8	1.1	3.6	3.6
53	**B**	4.3	14.2	3.5	1.8	3.5	7.1
Amp.^a^	-	**–**	**0.4**	**–**	**0.2**	**0.4**	**0.2**
Strept.^b^	-	**0.2**	**0.1**	**–**	**0.1**	**0.4**	**0.1**
Rif.^c^	-	**0.1**	**0.1**	**0.4**	**0.2**	**0.08**	**0.4**

^a^ Amp.—Ampicillin

^b^ Strept.—Streptomycin

^c^ Rif.—Rifampicin.

O—Orange type; B—Blue type of propolis,–not detected.

*All MIC values less than 1 mg/mL are bolded.

The sensitivity of Gram positive bacteria to propolis varied among the strains tested and the propolis samples used, however all of the strains tested were sensitive to all propolis samples. The strongest antibacterial activity was exhibited by sample **26** against *S*. *aureus* with a MIC value of 0.5 mg/mL and *L*. *monocytogenes* with a MIC as low as 0.1 mg/mL, which was also the lowest effective concentration observed in our study. Propolis samples **34**, **36, 44** and **46** also exhibited strong activity against all Gram positive bacteria, with MIC values from 0.3 to 2.2 mg/mL. All these samples belong to the orange type of propolis characterized by a higher content of phenolic compounds [7,8]. Overall, the most sensitive strain of Gram positive bacteria was *L*. *monocytogenes*, with MIC values from 0.1 to 1.9 mg/mL for orange and 0.4 to 10.6 mg/mL for blue propolis. The MIC values of propolis extracts from Greece and Cyprus tested on two *L*. *monocytogenes* strains, according to Kalogeropoulos et al. [16], were between 0.08 and 0.30 mg/mL, confirming that these strains are the most sensitive, as we found in the present study. Also, propolis samples collected from different geographical regions of Turkey showed antibacterial activities against *L*. *monocytogenes* [17]. However, this consistency does not exist with the rest of the Gram positive strains tested. For *B*. *subtilis*, *E*. *faecalis* and *S*. *aureus* the range of MIC was very broad, from 0.3 to well above 10 mg/mL, without observable grouping according to propolis type.

Regarding the sensitivity of the Gram negative bacteria tested, all propolis samples showed some effect, but on average MICs were higher compared to the sensitivity of Gram positive bacteria, although the range of MIC values was very broad (0.3–16.8 mg/mL). Grouping of propolis samples with respect to the two varieties was not observed. The lowest MIC of 0.3 mg/mL for *A*. *hydrophila*, obtained with samples **34** and **46**, was the lowest concentration that affected Gram negative bacteria. On the other hand, weak antimicrobial activity against Gram negative bacteria for samples **7**, **8**, **9**, **11**, **16**, **19**, **23**, **38** and **51** was shown, especially toward *A*. *hydrophila*. The antibacterial activity of propolis towards *A*. *hydrophila* has been confirmed by different authors [18,19], however MIC values were significantly higher in comparison to our study.

The most resistant strain tested in this study was *S*. *flexneri*, with MBC values above 10 mg/mL for forty seven propolis samples (S2 Table). Besides the strong effect of sample **26** (MIC of 2.0 mg/ml) on *S*. *flexneri*, only extracts **10** and **17** showed relatively low MICs, 1.8 mg/mL and 1.4 mg/mL, respectively. These results are in keeping with a study that found a strain from the genus *Shigella* (*S*. *dysenteriae*) to be the most resistant among all Gram negative bacteria tested [16]. But, recently Lou et al. [20] reported that *E*. *coli*, *Salmonella typhimurium* and *S*. *dysenteriae* were sensitive towards *p*-coumaric acid.

According to Pepeljnjak and Kosalec [21], samples of propolis with a high percentage of galangin have lower MIC and MBC values, while Mercan et al. [22] emphasize chrysin as an antimicrobial agent, whose high concentrations inhibit Gram negative bacteria. According to Ristivojević et al. [7,8], the phenolic compounds galangin, chrysin and pinocembrin were more abundant in the orange subgroup when compared to blue propolis. In line with this finding, our extracts that showed the weakest antimicrobial activity in the overall study were samples **5** and **15**, with MIC and MBC values higher than 12.0 mg/mL for most tested strains. These samples of propolis belong to the blue propolis type and their chemical composition revealed very low levels of phenolic compounds [7,8].

In general, the orange type of propolis shows higher antimicrobial activity in comparison to the blue type, probably due to the higher content of phenolic compounds. Orange propolis was observed to have statistically significantly higher activity (*t*_cr(one-tail)_ = 1.67 (*P* = 0.05, *d*.*f*. = 51)) when tested on *L*. *monocytogenes* (*t* = 5.57), *B*. *subtilis* (*t* = 3.94), *E*. *faecalis* (*t* = 2.37) and *S*. *flexneri* (*t* = 2.97).

Although the antimicrobial properties of propolis have been the subject of much research, it is difficult to compare the results of different studies, due to the varying composition of propolis extracts and the different methods used for the evaluation of propolis’ antibacterial activity [23]. Stepanović et al. [24] have previously analyzed the antimicrobial activity of propolis originating from Serbia and, without going into the chemical composition of the investigated samples, showed that the MIC values for Gram negative bacteria were higher in comparison to Gram positive strains. In general, numerous authors have shown that the antimicrobial effect of propolis is more pronounced toward Gram positive than Gram negative bacteria [25,26]. The structure of Gram positive bacteria cell wall, with predominant share of peptidoglycan, allows hydrophobic molecules to penetrate the cells and act on wall as well as cell membrane and within the cytoplasm. The cell wall of Gram negative bacteria is more complex with less peptidoglycan and with outer membrane composed of double layer of phospholipids linked with inner membrane by lipopolysaccharides [27]. Outer membrane is almost impermeable to hydrophobic molecules although some can slowly traverse trough porins [28,29]. Phenolic compounds generally show antimicrobial activity against Gram positive bacteria. Their effect depends on concentration; at low concentration they can interfere with enzymes of energy production and at higher concentration they can denature proteins [30]. But, Borges et al. [31] showed that phenolic acids such as ferulic and gallic acids, affect the cell membrane of both, Gram positive and Gram negative bacteria leading to a change in cell surface hydrophobicity and charge, consequently causing leakage of cytoplasmic content. The derivative of caffeic acid exhibited similar effect on cytoplasmic membrane of *Candida* cells [32].

### TLC fingerprint

Due to the prevalence of the orange type of propolis in Serbia and its higher observed antimicrobial activity, further chromatographic and multivariate evaluation was performed based only on the data obtained for this particular variety of propolis.

**Fig 1:**
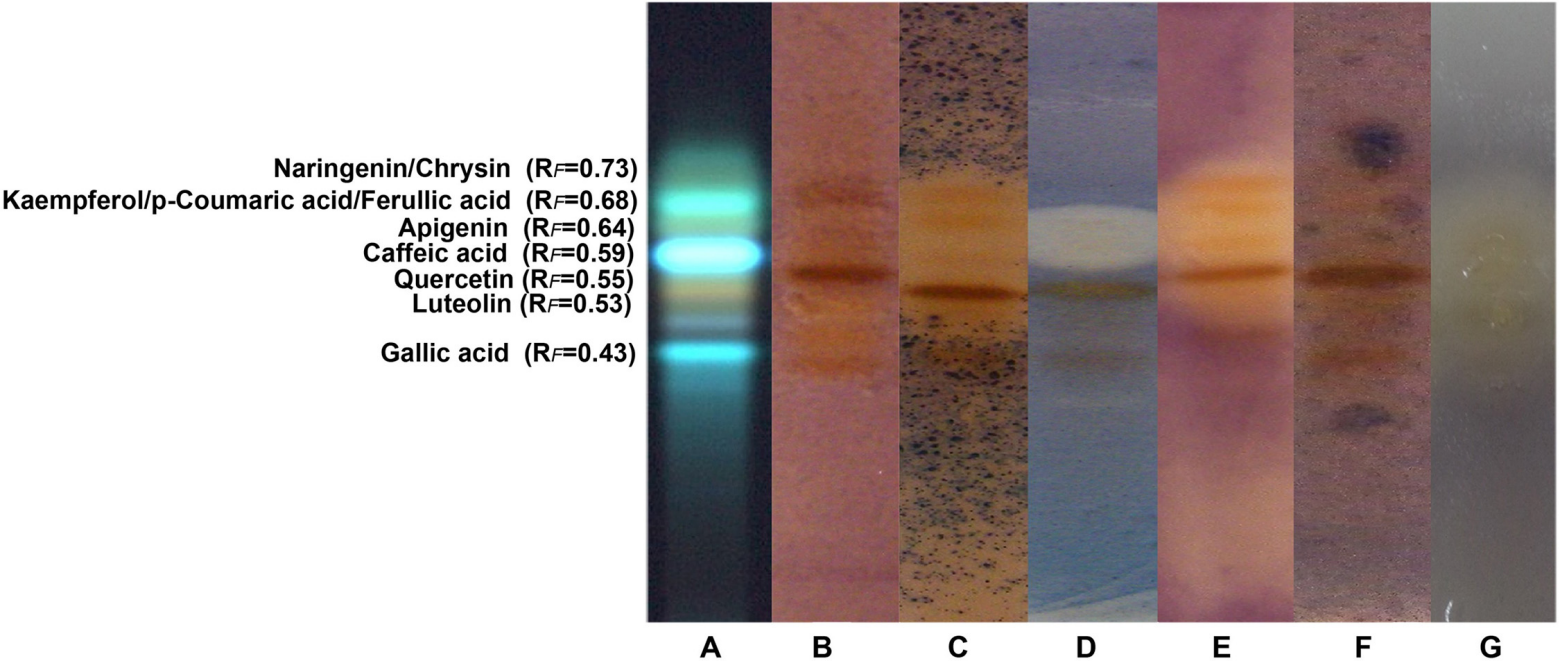
Bioautography assay of propolis samples against six bacterial strains. A) phenolic profile of propolis extract, B) *E*. *faecalis*, C) *B*. *subtilis*, D) *S*. *aureus*, E) *L*. *monocytogenes*, F) *A*. *hydrophila* and *G*) *S*. *flexneri*.

The antimicrobial activity of the orange variety of propolis was also estimated by TLC-indirect bioautography. TLC silica plates, developed according to the chromatographic conditions described in the previous section, were dipped in a suspension of analyzed microorganisms growing in an appropriate broth, thus making the surface of the plate a source of nutrients which enabled their growth [13]. The inhibition zones of microorganism growth formed as cream-white spots in the places where antimicrobial agents were present. A bioautography assay of each bacterium, together with the HPTLC chromatogram of one propolis sample (sample 26), are presented in Fig 1. All tested bacteria show different sensitivities to antimicrobial compounds present in the propolis samples, *i*.*e*. every strain gives a different bioautography profile. The antibacterial activity of propolis samples was associated with their most abundant components, compounds that appeared at *R*_F_ values from 0.40 to 0.70 (Fig 1).

Previously published results have revealed the influence of phenolics such as caffeic acid, galangin, quercetin, naringenin, pinostrombin, and chrysin on the antibacterial activity of propolis [18,21,33–35], similar to the results obtained in our study. The substances were associated with bands by comparing them with TLC chromatograms of standard phenolic compounds obtained under the same chromatographic conditions as the propolis extracts (S2 Fig). The influence of the minor compounds present in propolis, such as those at *R*_F_ values 0.53 and 0.55, should not be disregarded as they possess high activity against bacteria such as *B*. *subtilis*, *E*. *faecalis*, *S*. *aureus*, *L*. *monocytogenes* and *A*. *hydrophila*. Also, according to the bioautography assay for *S*. *aureus*, a synergistic effect between the compounds at *R*_F_ values 0.50 and 0.60 could be noticed.

In order to qualify the relationships between parameters that determine the antimicrobial activity of propolis, PLS modelling was performed on the HPTLC data set. For this purpose planar-chromatographic profiles obtained on amino-silica plates [7] were used due to the higher number of sharp bands, better separation efficiency and negligible background noise compared to non-modified silica gel. The HPTLC chromatogram of orange propolis [7], together with a chromatogram of a standard mixture, obtained on amino-silica plates to be comparable with the propolis phenolic profile, is presented in S1 Fig. Compounds such as caffeic acid (*R*_F_ = 0.37), quercetin/luteolin (*R*_F_ = 0.40), apigenin/*p*-coumaric acid/kaempferol (*R*_F_ = 0.45), naringenin/pinobanksin (*R*_F_ = 0.51), unknown compound 1 (*R*_F_ = 0.60) and unknown compound 2 at *R*_F_ value 0.70, were identified as characteristic markers of the orange variety of propolis.

The matrix used for PLS modelling consisted of 42 samples and 450 variables, expressed as the intensities values of pixels along the line segments obtained by digitization of the chromatograms. The resulting models could be used to indicate phenolic compounds that have the most influence on the antimicrobial activity of each propolis sample, but not for accurate prediction due to the unsatisfactory statistical quality of the model. The peaks potentially responsible for the antimicrobial activity of the propolis samples could be marked according to the regression coefficients of the resulting model [10, 11]. Chromatographic peaks with negative regression coefficients correspond to the compounds that exhibit antibacterial characteristics, as the MIC value decreases with increasing activity.

PLS models obtained for the relationship between antimicrobial activity (MIC, as the dependent variable) and HPTLC profile (*R*_F_ values, as the independent variable), for six bacterial strains separately, are summarized in Table 2. The number of latent variables (LVs) was selected on the basis of the minimum value of RMSECV, as well as the minimum difference between RMSEC and RMSECV values [10,11]. The peaks included in the final models for antimicrobial activity are presented in Table 2, in descending order of regression coefficients, with notification of the significance of their contribution to the dependent variable.

**Table 2:** Statistical parameters for the six PLS model.

Bacterial strain	LVs	RMSEC	RMSECV	Negative regression coefficient
*A*. *hydrophila*	3	0.3385	0.4166	Caffeic acid, quercetin/luteolin, and compounds at *R*_F_ 0.60 and 0.80
*S*. *flexneri*	2	0.2548	0.2939	Caffeic acid, apigenin/*p*-coumaric acid/kaempferol, naringenin/pinobanksin, compounds at *R*_F_ 0.70 and 0.80
*L*. *monocytogenes*	2	0.5677	0.6440	Apigenin/*p*-coumaric acid/kaempferol, naringenin/pinobanksin, and compounds at *R*_F_ 0.60
*S*. *aureus*	2	0.3501	0.3905	Caffeic acid, quercetin/luteolin, naringenin/pinobanksin and unknown compounds at *R*_F_ 0.60 and 0.70
*B*. *subtilis*	2	0.3501	0.3905	Apigenin/*p*-coumaric acid/kaempferol, naringenin/pinobanksin, unknown compounds at *R*_F_ 0.60 and 0.70
*E*. *faecalis*	2	0.3487	0.3990	Apigenin/*p*-coumaric acid/kaempferol, and compound at *R*_F_ 0.80

**Fig 2:**
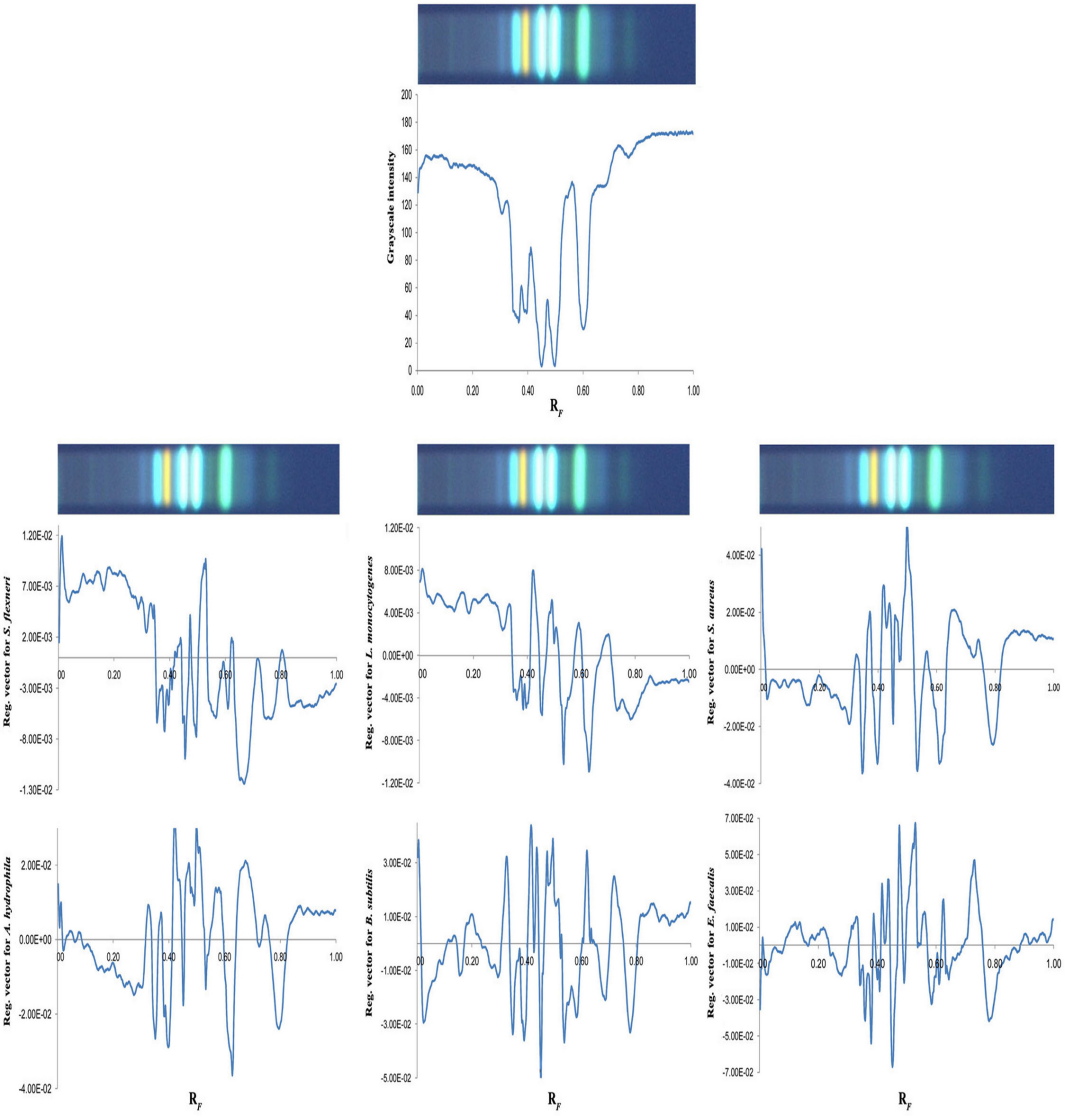
Regression coefficients of PLS models obtained from six bacterial strains.

The HPTLC fingerprint profile together with the line profile plots of chromatograms and a graph of the regression coefficients of PLS models are presented in Fig 2.

The most relevant peaks influencing the antimicrobial activity of propolis against all bacterial strains were the phenolic compounds at *R*_F_ values 0.37, 0.40, 0.45, 0.51, 0.60, 0.70 and 0.77. These bands have the highest negative values of regression coefficients, confirming the results of bioautography. Quercetin alone (the intense orange band at *R*_F_ value 0.40 on TLC chromatograms, Fig 2 and S1 Fig) significantly inhibited the growth of *S*. *aureus*, *B*. *subtilis* and *A*. *hydrophila* [32,34]. However, in combination with CAPE the activity of quercetin is reduced, suggesting antagonism which could be the reason for its decreased activity against *S*. *flexneri*, *L*. *monocytogenes* and *E*. *faecalis*. According to Boisard et al. [36], pinocembrin, pinobanksin-3-*O*-acetate, chrysin, and galangin, isolated from propolis extracts, exhibit moderate antibacterial activity against *S*. *aureus*. The PLS model for this bacterial strain distinguishes peaks at *R*_F_ values 0.37, 0.40, 0.51, 0.60 as dominant, followed by peaks at *R*_F_ values 0.45 and 0.77. The peak at *R*_F_ value 0.30 was also noticeable, and a peak at *R*_F_ value 0.60 obviously comprises several chromatographic bands, confirming the results of bioautography, the importance of low visible zones on TLC and the synergistic effect between components [37]. In the case of *B*. *subtilis*, the motility of bacteria in the presence of phenols was monitored and its inhibitory effect decreased as follows: CAPE > quercetin > naringenin > caffeic acid [38]. A regression plot obtained for *B*. *subtilis* revealed the importance of the peaks at *R*_F_ values 0.37 (caffeic acid), 0.45 (apigenin/*p*-coumaric acid/kaempferol, S1 Fig) and 0.51 (naringenin/pinobanksin), the link between peaks at *R*_F_ values 0.52 and 0.60, and the appearance of a new peak at *R*_F_ value 0.70 (not present in other bacterial strains), confirming previously published results. Compounds with antimicrobial activity against *A*. *hydrophila* were recognized as peaks at *R*_F_ values 0.37, 0.40, 0.60, and 0.77. According to the literature, quercetin (*R*_F_ = 0.40) and caffeic acid (*R*_F_ = 0.37) were identified as antimicrobial agents against *A*. *hydrophila* [33,39]. In addition, a series of overlapping peaks in the range of *R*_F_ values from 0.15 to 0.30 suggest the existence of a high number of compounds with low antimicrobial activity. Significant regression vectors for *E*. *faecalis* corresponded to bands at *R*_F_ values 0.45 and 0.77. Peaks with no clear discrimination were obtained for the range of *R*_F_ values from 0.37 to 0.40. Regression plots for *S*. *flexneri* and *L*. *monocytogenes* revealed the absence of antimicrobial compounds below *R*_F_ value 0.37, contrary to the *A*. *hydrophila* strain, and plenty of overlapping peaks in the remaining part of the chromatogram. According to the literature data [40,41] quercetin and its esters did not show any significant activity on *S*. *flexneri*, a finding which was also confirmed by an investigation of propolis samples. On the other hand, a previous publication [17,34,42] emphasized the high antimicrobial activity of caffeic acid and quercetin against *L*. *monocytogenes*, contrary to their moderate impact in Serbian propolis samples. According to Merkl et al. [43], phenolic acid and its alkyl esters are antimicrobial agents against *L*. *monocytogenes*, which is in agreement with our results.

## Conclusions

The utilization of HPTLC, MIC and bioautography for the analysis of complex mixtures of natural products, such as propolis, could be very useful. Its integration with chemometric techniques makes possible the identification of compounds potentially responsible for antibacterial capacity.

The antimicrobial activity of the Serbian propolis samples against various bacterial strains was determined by a MIC assay. It was confirmed that propolis samples with a certain combination of predominant phenolic compounds such as galangin, chrysin and pinocembrin are very effective against both Gram positive and Gram negative bacteria. The most sensitive strain of Gram positive bacteria was *L*. *monocytogenes*, while *B*. *subtilis*, *E*. *faecalis* and *S*. *aureus* strains showed wide range of MIC values.

The biological activity of the samples was further modelled as a function of HPTLC fingerprints using a PLS technique. The most relevant peaks influencing the antimicrobial activity of propolis against all bacterial strains were phenolic compounds such as caffeic acid, quercetin, luteolin, apigenin, *p*-coumaric acid, kaempferol, naringenin, pinobanksin, and two unknown compounds at *R*_F_ = 0.60 and 0.70. These bands have the highest negative values of regression coefficients. In addition, indirect bioautography was performed to localize the antibacterial activity on chromatograms. The results confirm phenolic compounds such as caffeic acid, quercetin, naringenin, and chrysin as the most active against observed bacteria.

The knowledge gained through this study could be important for attributing the antimicrobial activity of propolis to specific chemical compounds, as well as for verifying HPTLC fingerprints as a reliable method for the identification of compounds that are potentially responsible for antimicrobial activity. To the best of our knowledge, this is the first report on the multivariate regression modelling of antimicrobial activity of propolis as natural product and chemical composition obtained by HPTLC fingerprint in order to predict compounds with potential antimicrobial activity.

## Supporting Information

S1 Fig Standard phenolic compounds with orange type of propolis on NH_2_ HPTLC plate. The order was given from left to the right side: gallic acid, *p*-coumaric acid, caffeic acid, apigenin, luteolin, 2 orange propolis samples, naringenin, quercetin, kaempferol, pinostrombin, pinobanksin.
(TIF)

S2 Fig Standard phenolic compounds on HPTLC plate silica gel. The order was given from left to the right side: *p*-coumaric acid, caffeic acid, gallic acid, chlorogenic acid, ferullic acid, cinnamic acid, naringenin, quercetin, myricetin, kaempferol, rutin, luteolin, chrysin, apigenin.
(TIF)

S1 Table Information about the propolis samples, geographical coordinates, wax content and classification according to the HPTLC phenolic profile. (DOC)

S2 Table The minimum bactericidal concentration (MBC) of methanol extracts of Serbian propolis samples in mg/mL. (DOC)
