# Supplementary figures and images for: Antimicrobial Activity of Serbian Propolis Evaluated by Means of MIC, HPTLC, Bioautography and Chemometrics
Source: PLoS One. 2016 Jun 7;11(6):e0157097. doi: 10.1371/journal.pone.0157097 (PMC4896501; doi:10.1371/journal.pone.0157097)

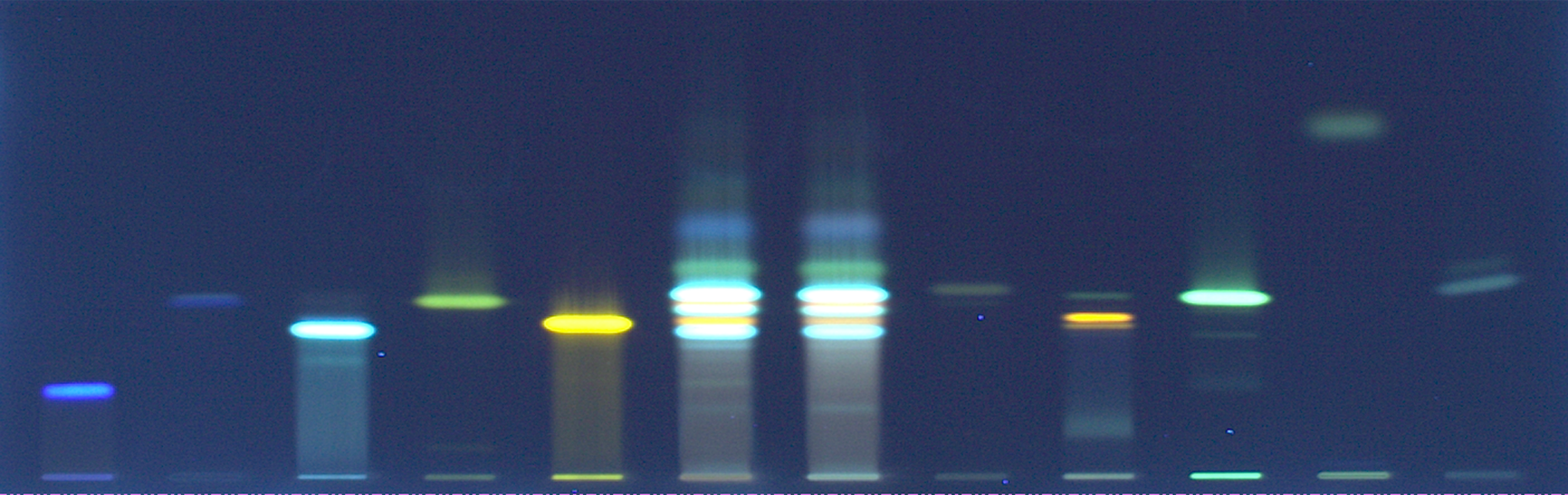

Supplement: S1 Fig — The order was given from left to the right side: gallic acid, p-coumaric acid, caffeic acid, apigenin, luteolin, 2 orange propolis samples, naringenin, quercetin, kaempferol, pinostrombin, pinobanksin. (TIF) [file pone.0157097.s001.tif]

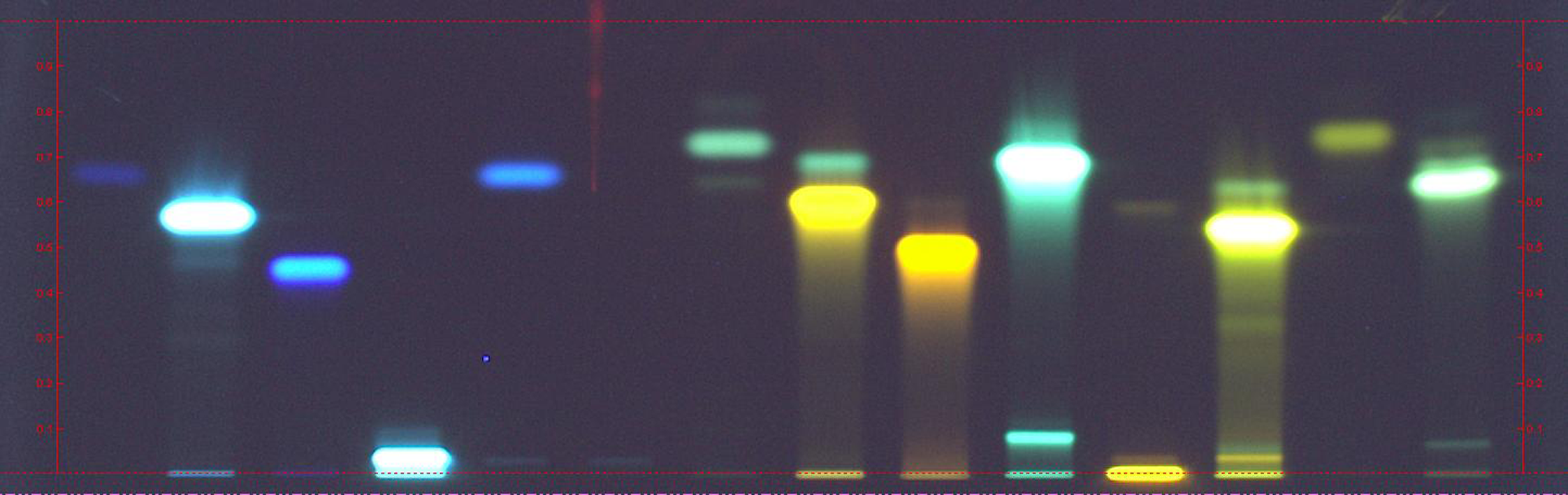

Supplement: S2 Fig — The order was given from left to the right side: p-coumaric acid, caffeic acid, gallic acid, chlorogenic acid, ferullic acid, cinnamic acid, naringenin, quercetin, myricetin, kaempferol, rutin, luteolin, chrysin, apigenin. (TIF) [file pone.0157097.s002.tif]
