# Supplementary material for: Antimicrobial Activity of Serbian Propolis Evaluated by Means of MIC, HPTLC, Bioautography and Chemometrics
Source: PLoS One. 2016 Jun 7;11(6):e0157097. doi: 10.1371/journal.pone.0157097 (PMC4896501; doi:10.1371/journal.pone.0157097)
Supplement: S1 Table — (DOC) [file pone.0157097.s003.doc]

**S1** Table

| **No.** | **Location** | **Location** | **Mass of sample (g)** | **Wax content %** | **Classification according to**  **HPTLC fingerprint** |
| --- | --- | --- | --- | --- | --- |
| 1 | West region - Serbia | 43°38′53″N 19°40′44″E | 1.01 | 61.39 | Blue |
| 2 | East region - Serbia | 44°22′42″N 21°25′10″E | 1.17 | 39.32 | Orange |
| 3 | Central region - Serbia | 44°00′40″N 20°54′40″E | 1.05 | 31.43 | Orange |
| 4 | West region - Serbia | 44°28′15″N 19°48′25.2″E | 1.00 | 29.00 | Blue |
| 5 | West region - Serbia | 44°28′15″N 19°48′25.2″E | 1.01 | 27.72 | Blue |
| 6 | South region - Serbia | 43°00′N 21°57′E | 1.01 | 33.66 | Orange |
| 7 | East region - Serbia | 44°14′1.1″N 21°44′49.54″E | 1.01 | 25.74 | Orange |
| 8 | East region - Serbia | 44°24′37″N 21°24′07″E | 1.01 | 24.75 | Orange |
| 9 | Vojvodina - Serbia | 45°7′0″N 21°18′12″E | 1.02 | 28.43 | Orange |
| 10 | Central region - Serbia | 43°23′31″N 21°26′57″E | 1.09 | 27.52 | Orange |
| 11 | Vojvodina - Serbia | 45°47′N 19°07′E | 1.02 | 29.41 | Orange |
| 12 | East region - Serbia | 45°23′0″N 20°22′54″E | 1.02 | 38.24 | Orange |
| 13 | West region - Serbia | 44°36′11″N 19°29′39″E | 1.01 | 24.75 | Orange |
| 14 | Belgrade region - Serbia | 44°49′N 20°28′E | 1.18 | 28.81 | Orange |
| 15 | Vojvodina - Serbia | 46°5′0″N 19°39′0″E | 0.99 | 35.35 | Blue |
| 16 | Vojvodina - Serbia | 46°5′0″N 19°39′0″E | 1.01 | 38.61 | Orange |
| 17 | Vojvodina - Serbia | 46°5′0″N 19°39′0″E | 1.04 | 45.19 | Orange |
| 18 | Vojvodina - Serbia | 46°6′0″N 19°34′0.12″E | 0.95 | 36.84 | Blue |
| 19 | Vojvodina - Serbia | 45°09′04″N 19°42′40″E | 1.02 | 31.37 | Orange |
| 20 | West region - Serbia | 44°22′N 20°10′E | 1.00 | 36.00 | Orange |
| 21 | Central region - Serbia | 44°18′N 20°34′E | 1.01 | 38.61 | Orange |
| 22 | South region - Serbia | 42°41′N 22°10′E | 1.01 | 26.73 | Orange |
| 23 | South region - Serbia | 42°41′N 22°10′E | 1.02 | 32.35 | Blue |
| 24 | South region - Serbia | 42°42′N 22°04′E | 1.01 | 22.77 | Orange |
| 25 | South region - Serbia | 42°42′N 22°04′E | 1.03 | 29.13 | Orange |
| 26 | South region - Serbia | 42°42′N 22°04′E | 0.99 | 19.19 | Orange |
| 27 | South region - Serbia | 42°42′N 22°04′E | 1.01 | 12.87 | Orange |
| 28 | Vojvodina - Serbia | 45°49′N 19°38′E | 1.04 | 21.15 | Orange |
| 29 | Vojvodina - Serbia | 45°14′12″N 19°37′23″E | 1.00 | 42.00 | Orange |
| 30 | Vojvodina - Serbia | 45°09′04″N 19°42′40″E | 1.02 | 19.61 | Orange |
| 31 | Vojvodina - Serbia | 45°12′N 19°56′E | 0.98 | 53.06 | Orange |
| 32 | Vojvodina - Serbia | 45°9′N 20°4′E | 1.08 | 52.78 | Orange |
| 33 | South region - Serbia | 42°52′0″N 21°55′0″E | 1.07 | 29.91 | Orange |
| 34 | South region - Serbia | 42°54′53″N 22°02′45″E | 1.04 | 29.81 | Orange |
| 35 | Vojvodina - Serbia | 45°07′N 19°14′E | 1.12 | 17.86 | Blue |
| 36 | Central region - Serbia | 43°43′25″N 20°41′15″E | 1.05 | 28.57 | Orange |
| 37 | West region - Serbia | 44°16′N 19°53′E | 1.03 | 9.71 | Orange |
| 38 | Vojvodina - Serbia | 43°43′N 21°22′E | 1.39 | 25.18 | Orange |
| 39 | Belgrade region - Serbia | 44°49′N 20°28′E | 1.09 | Without wax | Orange |
| 40 | South region - Serbia | 42°55′N 21°44′E | 1.05 | 15.24 | Orange |
| 41 | South region - Serbia | 42°52′0″N 21°55′0″E | 1.04 | 39.42 | Orange |
| 42 | Central region - Serbia | 43°22′53″N 21°2′16″E | 1.02 | 22.55 | Orange |
| 43 | Central region - Serbia | 43°22′53″N 21°2′16″E | 1.00 | 49.00 | Orange |
| 44 | Kosovo and Metohija - Serbia | 42°13′19″N 21°00′17″E | 1.04 | 11.54 | Orange |
| 45 | Kosovo and Metohija - Serbia | 42°13′19″N 21°00′17″E | 1.00 | 31.00 | Orange |
| 46 | Kosovo and Metohija - Serbia | 42°13′19″N 21°00′17″E | 1.08 | 15.74 | Orange |
| 47 | Kosovo and Metohija - Serbia | 42°13′19″N 21°00′17″E | 1.03 | 17.48 | Orange |
| 48 | East region - Serbia | 43°43′25″N 20°41′15″E | 1.07 | 15.89 | Blue |
| 49 | West region - Serbia | 44°16′N 19°53′E | 1.02 | 28.43 | Orange |
| 50 | Central region - Serbia | 43°35′N 21°19′E | 1.03 | 29.13 | Orange |
| 51 | West region - Serbia | 44°16′N 19°53′E | 1.01 | 34.65 | Orange |
| 52 | West region - Serbia | 44°16′N 19°53′E | 1.04 | 32.69 | Blue |
| 53 | Central region - Serbia | 44°00′40″N 20°54′40″E | 0.66 | 30.30 | Blue |
