# Supplementary material for: Antimicrobial Activity of Serbian Propolis Evaluated by Means of MIC, HPTLC, Bioautography and Chemometrics
Source: PLoS One. 2016 Jun 7;11(6):e0157097. doi: 10.1371/journal.pone.0157097 (PMC4896501; doi:10.1371/journal.pone.0157097)
Supplement: S2 Table — (DOC) [file pone.0157097.s004.doc]

| **S2 Table** | | | | | | | |
| --- | --- | --- | --- | --- | --- | --- | --- |
| **Extracts of propolis** | **Type of propolis** | ***Aeromonas hydrophila* ATCC 49140** | ***Shigella flexneri***  **ATCC 9198** | ***Listeria monocytogenes* ATCC 19111** | ***Staphylococcus aureus***  **ATCC 25922** | ***Bacillus subtilis***  **ATCC 6632** | ***Enterococcus faecalis***  **ATCC 29212** |
| 2 | **O** | 6.1 | 12.1 | 0.8 | 12.1 | 3.0 | 12.1 |
| 3 | **O** | 13.7 | 13.7 | 1.7 | – | 6.9 | 13.7 |
| 6 | **O** | – | 6.6 | 13.3 | – | 13.3 | 3.3 |
| 7 | **O** | – | 14.9 | 14.9 | 14.9 | – | 3.7 |
| 8 | **O** | – | 15.1 | 15.1 | 12.0 | 15.1 | 3.8 |
| 9 | **O** | 14.3 | 14.3 | 14.3 | 14.3 | 14.3 | 3.6 |
| 10 | **O** | 14.5 | 3.6 | 14.5 | – | 3.6 | 14.5 |
| 11 | **O** | – | 7.1 | 14.1 | 8.5 | 14.1 | 3.5 |
| 12 | **O** | – | – | – | – | – | 12.4 |
| 13 | **O** | 7.5 | 15.0 | 1.9 | 1.1 | 7.5 | 3.8 |
| 14 | **O** | 7.2 | 14.2 | 3.6 | – | 14.2 | 3.6 |
| 16 | **O** | – | – | – | – | – | 3.1 |
| 17 | **O** | – | – | – | – | – | 1.4 |
| 19 | **O** | – | 13.7 | – | – | – | 13.7 |
| 20 | **O** | 6.4 | 12.8 | 3.2 | 3.2 | – | 3.2 |
| 21 | **O** | 6.1 | 12.3 | 6.1 | 1.5 | – | 3.1 |
| 22 | **O** | 8.5 | – | 3.6 | 3.6 | – | 14.7 |
| 24 | **O** | 3.1 | 15.4 | 1.9 | 1.0 | 7.7 | 3.9 |
| 25 | **O** | 1.5 | 14.2 | 3.5 | 1.8 | 3.5 | 7.1 |
| 26 | **O** | 4.0 | 4.0 | 1.0 | 2.0 | 4.0 | 4.0 |
| 27 | **O** | 17.4 | 17.4 | 4.4 | 2.2 | 17.4 | 17.4 |
| 28 | **O** | 15.8 | 15.8 | 15.8 | – | – | – |
| 29 | **O** | – | – | 11.6 | – | – | – |
| 30 | **O** | 16.1 | 16.1 | 16.1 | – | 16.1 | 4.0 |
| 31 | **O** | 2.4 | 9.4 | 1.2 | 1.2 | 9.4 | 4.7 |
| 32 | **O** | 9.4 | 9.4 | – | – | – | 2.4 |
| 33 | **O** | 1.8 | 14.0 | 1.8 | 1.8 | 3.5 | 6.9 |
| 34 | **O** | 1.9 | 14.0 | 0.5 | 0.9 | 3.5 | 3.5 |
| 36 | **O** | 7.1 | 14.3 | 0.9 | 0.9 | – | 1.8 |
| 37 | **O** | 9.0 | 18.0 | 1.2 | 2.3 | 18.0 | 4.5 |
| 38 | **O** | 15.0 | – | 1.0 | 1.8 | – | 7.5 |
| 40 | **O** | 3.8 | 17.0 | 1.3 | 1.1 | 4.2 | 8.5 |
| 41 | **O** | 1.2 | 12.1 | 3.0 | 1.5 | 1.5 | 12.1 |
| 42 | **O** | 1.9 | 15.5 | 1.9 | 1.0 | 3.9 | 7.8 |
| 43 | **O** | 3.6 | 10.2 | 1.3 | 0.6 | 5.1 | 2.6 |
| 44 | **O** | 1.7 | 17.7 | 2.2 | 1.1 | 8.9 | 8.9 |
| 45 | **O** | 1.3 | 13.8 | 3.5 | 1.7 | 1.7 | 3.5 |
| 46 | **O** | 1.5 | 16.9 | 4.2 | 1.1 | 2.1 | 8.4 |
| 47 | **O** | 6.6 | 16.5 | 0.8 | 1.0 | 4.1 | 4.1 |
| 49 | **O** | 4.2 | – | 0.5 | 4.2 | 8.5 | 8.5 |
| 50 | **O** | 7.7 | – | 0.8 | 1.9 | 3.8 | 7.7 |
| 51 | **O** | – | – | 16.8 | 5.9 | 16.8 | – |
| 1 | **B** | 7.7 | 6.1 | 3.9 | 7.7 | 7.7 | 7.7 |
| 4 | **B** | 14.2 | 14.2 | 14.2 | – | 12.8 | 14.2 |
| 5 | **B** | – | – | 14.5 | – | 13.0 | 7.2 |
| 15 | **B** | – | – | 12.9 | – | – | – |
| 18 | **B** | 6.3 | 12.6 | 6.3 | 3.2 | – | 6.3 |
| 23 | **B** | 13.5 | – | 3.4 | 6.8 | – | 6.8 |
| 35 | **B** | 8.2 | 16.4 | 16.4 | 8.2 | 16.4 | 16.4 |
| 39 | **B** | 10.0 | – | 4.8 | 2.6 | 20.0 | 10.0 |
| 48 | **B** | 5.3 | – | – | 10.6 | – | – |
| 52 | **B** | 14.3 | – | 7.2 | 1.8 | 14.3 | 7.2 |
| 53 | **B** | 8.5 | – | 7.1 | 3.5 | 14.2 | 14.2 |
| Amp.^a^ | - | – | 1.8 | – | 0.6 | 1.4 | 0.6 |
| Strept.^b^ | - | 0.3 | 0.4 | – | – | – | 0.4 |
| Rif.^c^ | - | – | 0.4 | – | 0.4 | 0.2 | – |

^a^ Amp. - Ampicillin; ^b^ Strept. - Streptomycin; ^c^ Rif. - Rifampicin.

O - Orange type; B - Blue type of propolis, **–** not detected.
